# Consistent Inverse Associations of Total, “Bioavailable”, Free, and “Non-Bioavailable” Vitamin D with Incidence of Diabetes among Older Adults with Lower Baseline HbA_1c_ (≤6%) Levels

**DOI:** 10.3390/nu14163282

**Published:** 2022-08-11

**Authors:** Anna Zhu, Sabine Kuznia, Tobias Niedermaier, Bernd Holleczek, Ben Schöttker, Hermann Brenner

**Affiliations:** 1 Division of Clinical Epidemiology and Aging Research, German Cancer Research Center (DKFZ), 69120 Heidelberg, Germany; 2 Medical Faculty Heidelberg, Heidelberg University, 69120 Heidelberg, Germany; 3 Saarland Cancer Registry, 66119 Saarbrücken, Germany; 4 Network Aging Research, Heidelberg University, 69115 Heidelberg, Germany; 5 Division of Preventive Oncology, German Cancer Research Center (DKFZ) and National Center for Tumor Diseases (NCT), 69120 Heidelberg, Germany; 6 German Cancer Consortium (DKTK), German Cancer Research Center (DKFZ), 69120 Heidelberg, Germany

**Keywords:** vitamin D, vitamin D-binding protein, bioavailable 25(OH)D, free 25(OH)D, type 2 diabetes

## Abstract

Background: Serum 25-hydroxyvitamin (25(OH)D) levels are inversely associated with risk of diabetes. The “free hormone hypothesis” suggests potential effects to be mainly related to concentrations of “bioavailable” and free rather than total 25(OH)D. We assessed associations of serum concentrations of vitamin D-binding protein (VDBP), as well as total “bioavailable”, complementary “non-bioavailable”, and free 25(OH)D, with the risk of developing diabetes among non-diabetic older adults in a large population-based cohort study in Germany. Methods: We included 4841 non-diabetic older adults aged 50–75 years at the baseline exam from the ESTHER cohort conducted in Saarland, Germany, in 2001–2002. Concentrations of “bioavailable” and free 25(OH)D were derived from serum concentrations of VDBP, total 25(OH)D, and albumin. Incidence of diabetes was ascertained during up to 14 years of follow-up. Associations were quantified by multivariable Cox proportional hazards regression models with comprehensive confounder adjustment. Results: During a median follow-up of 10.6 years, 837 non-diabetic participants developed diabetes. We observed similar inverse associations with developing diabetes for VDBP (hazard ratio (HR) for lowest versus highest quintile: 1.37, 95% confidence interval (CI): 1.09, 1.72), total 25(OH)D (HR: 1.31, 95% CI: 1.03, 1.66), and “non-bioavailable” 25(OH)D (HR: 1.30, 95% CI: 1.02, 1.65). Associations were smaller and statistically insignificant for “bioavailable” and free 25(OH)D. However, associations of total “non-bioavailable”, “bioavailable”, and free 25(OH)D with incidence of diabetes were much stronger among, and essentially restricted to, participants with lower baseline HbA_1c_ (≤6%) levels. Conclusions: This large prospective cohort study of older Caucasian adults, in agreement with results from randomized trials and Mendelian randomization studies, supports a protective effect of vitamin D against development of diabetes. The “free hormone theory” may not be relevant in this context. However, our results underline the importance of adequate vitamin D status among those who have not yet shown any sign of impaired glucose tolerance.

## 1. Introduction

An increasing proportion of the world population is living with type 2 diabetes; the global prevalence is expected to rise from approximately 6% in 2017 to more than 7% in 2030 [1]. Observational epidemiological studies have consistently identified vitamin D deficiency as a risk factor for type 2 diabetes [2]. Most but not all recent Mendelian randomization studies have supported causality of this association [3,4,5,6]. A meta-analysis of randomized, controlled trials found that vitamin D supplementation reduced the risk of type 2 diabetes [7]. 

Previous epidemiological evidence of the association between vitamin D status and diabetes risk has almost exclusively been based on studies that defined vitamin D status according to 25-hydroxyvitamin D (25(OH)D). Other biomarkers of vitamin D status, such as “bioavailable” or free (25(OH)D) have been suggested to be better indicators of vitamin-D-related health outcomes. Approximately 85–90% of serum 25(OH)D is bound to vitamin D-binding protein (VDBP), whereas approximately 10–15% is loosely bound to albumin [8], with the remainder (<1%) freely circulating. Albumin-bound 25(OH)D, together with free 25(OH)D, has been labelled “bioavailable” 25(OH)D [9,10]. The “free hormone hypothesis” suggests that the biological activity of a hormone is defined by the concentration of its free form that can easily diffuse through cell membranes. This hypothesis has not been consistently verified for vitamin D and diabetes [11]. We aimed to comprehensively evaluate associations of VDBP, as well as total, “bioavailable”, complementary “non-bioavailable”, and free 25(OH)D with the risk of developing type 2 diabetes among non-diabetic older adults in a large population-based cohort in Germany. 

## 2. Methods

### 2.1. Study Design and Population

This study is based on data from the ESTHER study (German name: Epidemiologische Studie zu Chancen der Verhütung, Früherkennung und optimierten Therapie chronischer Erkrankungen in der älteren Bevölkerung). ESTHER is a large-scale prospective cohort study conducted in Saarland, Germany, that was established to explore novel approaches to prevention and early detection of chronic diseases among older adults. More details on the study design can be found elsewhere [12]. Briefly, between 2000 and 2002, 9940 women and men aged 50–75 years were recruited by their general practitioners (GPs) during a routine health checkup. Follow-up data collections, including participant and GP questionnaires and a comprehensive mortality follow-up through population registries, were conducted 2, 5, 8, 11, and 14 years after recruitment.

Our study focuses on participants who were recruited between 2001 and 2002, had available serum concentrations of VDBP, total 25(OH)D, and albumin, as well as genetic markers for deriving “bioavailable” and free 25(OH)D concentrations [13]. We further restricted the analysis to nondiabetic participants with available incidence data for type 2 diabetes during the follow-up. 

### 2.2. Data and Blood Sample Collection

Data on sociodemographic and lifestyle factors, family, and medical history of diabetes were collected in considerable detail from standardized questionnaires from both participants and their GPs. Systolic blood pressure, height, and weight were measured by GPs. Blood samples were taken, centrifuged, and shipped to the study center, where they were stored at −80 °C until analysis. Serum concentrations of albumin were measured by fluorescence immunoassay; C-reactive protein (CRP) by turbidimetry; creatinine by a kinetic Jaffé method; and high-density lipoprotein (HDL), cholesterol, and total cholesterol by enzymatic chromatography. The creatinine-based Chronic Kidney Disease Epidemiology Collaboration equation was applied to calculate the glomerular filtration rate (eGFR) [14].

### 2.3. Vitamin D Measurements

Measurement and standardization of serum total 25(OH)D were performed as previously reported [15] and outlined in the Supplementary Methods. In brief, we measured total 25(OH)D concentrations separately by sex in the context of two research projects. In 2006, a Diasorin-Liaison analyzer (Diasorin Inc., Stillwater, OK, USA) was used to measure total 25(OH)D concentrations among women. In 2009, an IDS-iSYS instrument (Immunodiagnostic Systems GmbH, Frankfurt Main, Germany) was applied to measure total 25(OH)D concentrations among men because the Diasorin-Liaison method was no longer available. We standardized both immunoassays to the gold-standard method of liquid chromatography tandem mass spectrometry as previously described [15]. 

In 2019, we measured serum VDBP concentrations by using a polyclonal enzyme immunoassay (Immundiagnostik Inc., Bensheim, Germany). The intra-assay and inter-assay coefficients of variations for the samples were less than 10%. Array-based genotyping was performed using an Illumina Infinium OncoArray and Global Screening Array BeadChips (Illumina, San Diego, CA, USA). More information on quality control assessment and imputation of genetic data has been reported in detail elsewhere [16] and can be found in the Supplementary Methods. Genetic data of single nucleotide polymorphism (SNP) rs7041 and rs4588 were extracted to code VDBP genotypes. The coding of VDBP genotypes is presented in Supplementary Table S1.

Free 25(OH)D and “bioavailable” 25(OH)D concentrations were derived from total 25(OH)D, VDBP, albumin concentrations, and their affinity constants derived from the VDBP genotypes with the following equations [17,18]:

$$D_{\mathrm{free}}=(-b+\sqrt{b^{2}-4\mathrm{ac}})\;÷\;2a,$$

where a = K_VDBP_ · K_alb_ · D_alb_ + K_VDBP_; b = K_VDBP_ · D_VDBP_ − K_VDBP_ · D_total_ + K_alb_ · D_alb_ + 1; and c = −(D_total_), and

D_”bioavailable”_ = D_free_ + D_alb_ = (K_alb_ · D_alb_ + 1) · D_free_,

where D_alb_ indicates albumin concentrations, D_”bioavailable”_ indicates “bioavailable” 25(OH)D concentrations, D_free_ indicates free 25(OH)D concentrations, D_total_ indicates total 25(OH)D concentrations, D_VDBP_ indicates VDBP concentrations, K_alb_ is the affinity constant between vitamin D and albumin (K_alb_ = 6 × 10^5^ M^−1^), and K_VDBP_ is the affinity constant between vitamin D and VDBP (K_VDBP_ = 1.12 × 10^9^ M^−1^ for GC1f-1f; K_VDBP_ = 8.6 × 10^8^ M^−1^ for GC1f-1s; K_VDBP_ = 7.4 × 10^8^ M^−1^ for GC1f-2; K_VDBP_ = 6.0 × 10^8^ M^−1^ for GC1s-1s; K_VDBP_ = 4.8 × 10^8^ M^−1^ for GC1s-2; and K_VDBP_ = 3.6 × 10^8^ M^−1^ for GC2-2). All concentrations are expressed in mol/L in calculating equations.

We defined “non-bioavailable” 25(OH)D concentrations as the difference between total and “bioavailable” 25(OH)D concentrations [13]. The term “non-bioavailable” 25(OH)D was created solely to make it clear that it is the complementary 25(OH)D that is not included in the common definition of “bioavailable” 25(OH)D, and it should not be interpreted to indicate lack of biological function. 

### 2.4. Diabetes Ascertainment

We ascertained the incidence of diabetes by GP-confirmed patient self-reports, prescribed drugs (Anatomical Therapeutic Chemical Classification code A10), GP records, or measurement of HbA_1c_ in blood samples obtained at follow-ups (defining HbA_1c_ ≥ 6.5% (48 mmol/mol) as new cases), as previously described in detail [19]. 

### 2.5. Statistical Analysis

We used descriptive statistics to summarize the baseline characteristics of included participants. We compared the baseline characteristics between participants who developed diabetes during the follow-up and those who did not. Chi-square (for categorical variables) or Kruskal–Wallis (for continuous variables) tests were applied to identify group differences.

We conducted multiple imputation to account for missing values (assumed to be random) in the covariates, including education; smoking; alcohol consumption; multivitamin supplement intake; vegetable, fruit, and fish consumption; physical activity; body mass index (BMI); CRP; HbA_1c_; HDL cholesterol; triglycerides; systolic blood pressure; family history of diabetes; antihypertensive medication; and lipid-lowering medication. In total, 20 imputed databases were generated from the imputation, which were further pooled together for all regression analyses. 

We applied Cox proportional hazards regression models to evaluate associations of various vitamin D biomarkers with diabetes incidence. We quantified associations of vitamin D biomarkers, which were entered in the models either as categorical variables (by quintiles) or as continuous variables, with diabetes incidence by hazard ratios (HRs) and 95% confidence intervals (CIs). Two types of regression models with various levels of covariate adjustment were run. Model 1 adjusted for age; sex; education; smoking; alcohol consumption; vegetable, fruit, and fish consumption; regular intake of multivitamin supplements; BMI; and season of blood draw. Model 2 additionally adjusted for HbA_1c_, total cholesterol, HDL cholesterol, triglycerides, CRP, systolic blood pressure, estimated GFR, family history of diabetes, history of cardiovascular diseases and cancer, antihypertensive medication, and lipid-lowering medication. In addition to analyses in the entire cohort, we conducted analyses in subgroups defined by age, sex, BMI, season of blood draw, family history of diabetes, baseline HbA_1c_ concentrations, history of cardiovascular disease and cancer, and baseline total 25(OH)D levels. We also tested the statistical significance of interactions between those characteristics and vitamin D biomarkers. We explored dose–response relationships of vitamin D biomarker concentrations with the risk of developing diabetes by plotting restricted cubic splines with knots at the 25th, 50th, and 75th percentiles (as the reference) [20]. Furthermore, we plotted cumulative incidence of diabetes over the 14-year follow-up according to vitamin D biomarker concentrations (above or below the median). All analyses were conducted with R software (version: 3.6.2, R Core Team, R Foundation for Statistical Computing, Vienna, Austria). Statistical significance was defined as *p* < 0.05 in two-sided testing.

## 3. Results

In total, 4841 nondiabetic older adults were included (Table 1). The mean age was 61.9 (standard deviation (SD): 6.6) years, and 42.5% of participants were male. Those who developed diabetes during the follow-up were, on average, slightly younger, less educated, and current smokers. They more commonly had a family history of diabetes, a history of cardiovascular disease, and used antihypertensive and lipid-lowering medication. They also had higher mean BMI, HbA_1c_, triglycerides, CRP, systolic blood pressure, and estimated GFR but lower HDL cholesterol. 

During a median follow-up of 10.6 years, 837 non-diabetic participants developed diabetes. In the most comprehensively adjusted model (2), the lowest quintile of VDBP concentrations was associated with a 37% (95% CI: 9–72%) increased rate of developing diabetes compared with the highest quintile (Table 2). A slightly weaker increase was observed for total (31%, 95% CI: 3–66%) and “non-bioavailable” 25(OH)D (30%, 95% CI: 2–65%). One SD decrease in VDBP concentrations was associated with a 9% (95% CI: 1–18%) increased rate of developing diabetes. The same increase (9%) was observed for total (95% CI: 0–18%) and “non-bioavailable” 25(OH)D (95% CI: 0–18%). The associations of “bioavailable” and free 25(OH)D concentrations with incidence of diabetes were weaker and not statistically significant.

Supplementary Figure S1 shows cumulative incidences of diabetes according to vitamin D biomarker concentrations. They were consistently somewhat higher for those with biomarker levels below the median than among those with biomarker levels above the median throughout the 14 years of follow-up for all vitamin D biomarkers.

Results of the subgroup analyses are shown in Table 3. Associations of vitamin D biomarkers with diabetes incidence did not significantly vary by baseline total 25(OH)D status, age, sex, BMI, season of blood draw, family history of diabetes, history of cardiovascular disease or cancer, or baseline 25(OH)D level. However, associations of total, “non-bioavailable”, “bioavailable”, and free 25(OH)D with incidence of diabetes were much stronger among, and essentially restricted to, participants with lower baseline HbA_1c_ (≤6%) levels. All interaction tests between HbA_1c_ and these vitamin D biomarkers were statistically significant. No interaction was observed between VDBP and baseline HbA_1c_.

**Figure 1:**
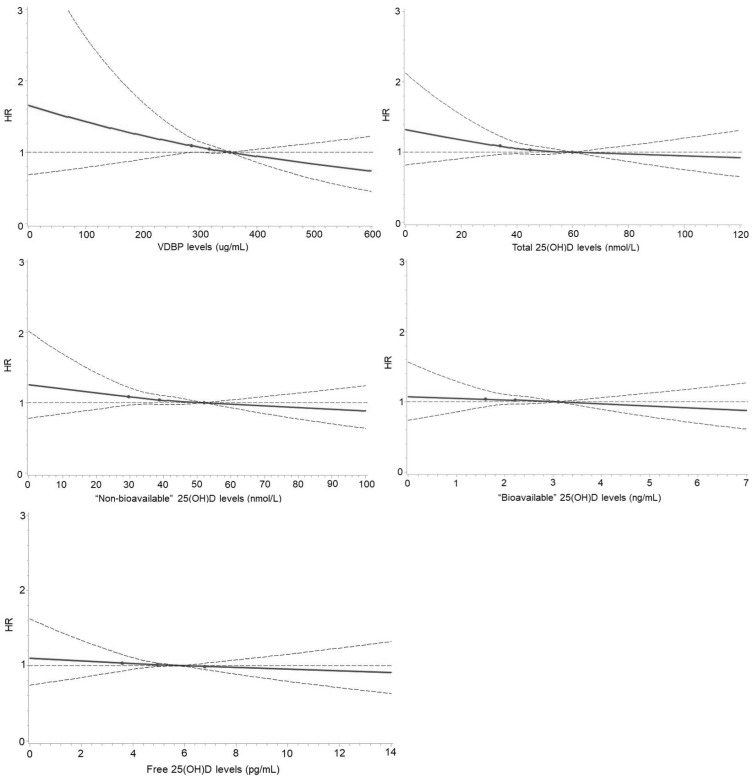
Dose–response curves for the associations of VDBP, total, “non-bioavailable”, “bioavailable”, and free 25(OH)D concentrations with risk of developing diabetes. Curves were derived using restricted cubic splines with three knots at 25, 50, and 75 (as the reference) percentiles of VDBP, total, “non-bioavailable”, “bioavailable”, and free 25(OH)D concentrations. Associations were multivariable-adjusted for age; sex; education; smoking and drinking status; vegetable, fruit, and fish consumption; regular intake of multivitamin supplements; body mass index; hemoglobin A1c; total cholesterol; high-density lipoprotein cholesterol; triglycerides; C-reactive protein; systolic blood pressure; estimated glomerular filtration rate; family history of diabetes; history of cardiovascular diseases and cancer; antihypertensive medication; lipid-lowering medication; and season of blood draw. There were 27 (0.6%) participants with VDBP > 600 µg/mL; 62 (1.3%) with total 25(OH)D > 120 nmol/L; 84 (1.7%) with “non-bioavailable” 25(OH)D > 100 nmol/L; 62 (1.3%) with “bioavailable” 25(OH)D > 7 ng/mL; and 105 (2.2%) with free 25(OH)D > 14 pg/mL.

Figure 1 shows the results of the dose–response analyses. Whereas monotonic inverse relationships were observed between VDBP, total and “non-bioavailable” 25(OH)D and incident diabetes, no clear relationships were observed for “bioavailable” and free 25(OH)D.

## 4. Discussion

Although multiple studies have assessed the association of total 25(OH)D with diabetes incidence, evidence of the specific contributions of VDBP, “bioavailable”, free, and “non-bioavailable” 25(OH)D to the prediction of diabetes risk has remained sparse and is considerably expanded by our study. Our large prospective cohort study of older adults from Germany revealed a clear inverse association between VDBP, total, and “non-bioavailable” 25(OH)D levels and incidence of diabetes during 14 years of follow-up. However, associations with diabetes were weaker (and not statistically significant) for “bioavailable” and free 25(OH)D than for total and “non-bioavailable 25(OH)D”, suggesting that the free hormone hypothesis may not be relevant to type 2 diabetes in this Caucasian population. Consistent strong associations between total, “non-bioavailable”, “bioavailable”, and free 25(OH)D and incidence of diabetes were observed among participants with lower baseline HbA_1c_ (≤6%) levels.

Based on the free hormone hypothesis, “bioavailable” or free 25(OH)D would be expected to be more biologically active than “non-bioavailable” 25(OH)D, that is, bound to albumin or VDBP [21]. However, this hypothesis does not seem to be supported by epidemiological evidence with respect to diabetes-related outcomes. Consistent with our study, a large cross-sectional study among 1904 health workers in Mexico suggested that free and “bioavailable” 25(OH)D do not provide incremental values for prediction of adiposity and several metabolic traits compared with total 25(OH)D [22]. Although plasma free 25(OH)D levels were more strongly associated with insulin resistance than plasma total 25(OH)D levels in a cross-sectional study among 1189 non-diabetic Hispanics and African Americans from the United States, the difference in risk estimates between total and free 25(OH)D was modest. No marked differences were observed between Hispanics and African Americans [23]. Likewise, in a cross-sectional study among Aboriginal Canadians, lower levels of “bioavailable” and total 25(OH)D were associated with increased insulin resistance and reduced β-cell function [24]. Overall, there is little if any support for the relevance of the free hormone hypothesis with respect to outcomes related to type 2 diabetes.

Some previous examinations reported stronger associations with various health endpoints, including total mortality, for “bioavailable” or free 25(OH)D than for total 25(OH)D [25,26]. However, these studies were conducted in specific and partial rather small patient cohorts. In a previous analysis of our large ESTHER cohort, we did not find evidence for superior prediction of total or cause-specific mortality by “bioavailable” or free compared to total 25(OH)D [27]. Nevertheless, apparent differences between studies may also be partly due to differences in other important factors, such as ethnicity of study populations, which require further study.

Interestingly, associations between total, “non-bioavailable”, “bioavailable”, and free 25(OH)D and incidence of diabetes were much stronger among, and essentially restricted to, participants with lower baseline HbA_1c_ (≤6%) levels in our study. This finding is consistent with results of recent studies from China and the United States, which reported inverse associations between serum 25(OH)D levels and the risk of developing prediabetes among healthy adults [28,29]. Potential mechanisms, which require further study, might include vitamin-D-associated stimulation of insulin secretion in pancreatic β-cells and the expression of the insulin receptor to improve insulin responsiveness for glucose transport [30,31]. Additionally, vitamin D can reduce inflammation and maintain Ca^2+^ levels to reduce risks of insulin resistance [32,33].

Our finding of an inverse association between VDBP concentrations and risks of developing type 2 diabetes among nondiabetic older adults is consistent with and strongly expands the limited evidence of this association reported in other studies. In a cross-sectional analysis based on the Canadian Multicentre Osteoporosis Study, which included 2254 men and women, an inverse association was observed between VDBP concentrations with fasting glucose levels and risk of type 2 diabetes [34]. Further evidence is mostly based on much smaller, predominantly cross-sectional studies. A study consisting of 236 healthy overweight and obese women from Iran showed that higher VDBP concentrations were associated with lower levels of insulin resistance [35]. A study of 47 postmenarchal female adolescents from the United States reported that VDBP concentrations were negatively correlated with fasting insulin levels [36]. A case–control study of 88 adults from India showed that individuals with type 2 diabetes had significantly lower VDBP levels than controls [37]. Similar findings were observed in another two case-control studies from the United States [38] and Saudi Arabia [39]. However, a study of 90 women with polycystic ovary syndrome from Australia showed no significant correlation between VDBP concentrations and insulin resistance [40].

Evidence from animal studies provided plausible explanations for the inverse association of VDBP concentrations and type 2 diabetes. VDBP can regulate the α-cell phenotype, leading to smaller and hyperplastic α cells, more F-actin microfilaments, changes in Na^+^-channel conductance, α-cell activation impairment, and decreased glucagon secretion, which further affects diabetes pathogenesis [41]. VDBP can also regulate the amount of active vitamin D in β-cells of the pancreas to influence insulin secretion [36].

Strengths of our study include the prospective cohort design, the very large sample size, the long-term follow-up with comprehensive ascertainment of incident diabetes through multiple data sources, side-by-side assessment of multiple vitamin D biomarkers (VDBP, total, “non-bioavailable”, “bioavailable”, and free vitamin D), and comprehensive adjustment for potential confounders, as well as comprehensive subgroup, interaction, and dose–response analyses. However, a number of limitations also need to be noted. Although numerous potential confounders were considered and adjusted for in the regression models, we cannot rule out potential residual confounding. Similar to most other studies, vitamin D biomarkers were measured only at baseline. Their potential changes over time could therefore not be considered and may have led to underestimation of associations due to (presumably nondifferential) imprecision of exposure ascertainment. “Bioavailable” and free 25(OH)D were derived from VDBP, total 25(OH)D, and albumin concentrations. Concentrations derived this way are thought to be higher than direct measurements, especially under specific physiologic and pathologic conditions [42]. Furthermore, our results with respect to this Caucasian population may not be generalized to other ethnic groups, as associations of VDBP phenotypes and type 2 diabetes have been found to vary between ethnic groups [11].

## 5. Conclusions

In this large prospective cohort study, lower VDBP levels, as well as total and non-bioavailable 25(OH)D concentrations, were associated with increased risk of type 2 diabetes among non-diabetic older adults. These associations persisted after comprehensive confounder adjustment. However, associations with diabetes were weaker and not statistically significant for “bioavailable” and free 25(OH)D concentrations, suggesting that the “free hormone hypothesis” may not be relevant with respect to type 2 diabetes in this Caucasian population. However, associations of total, non-bioavailable, bioavailable, and free 25(OH)D with incidence of diabetes were much stronger among, and essentially restricted to, participants with lower baseline HbA_1c_ (≤6%) levels, indicating the importance of adequate vitamin D status among those who have not yet shown any sign of impaired glucose tolerance. Potential differences in predicting risks of diabetes between specific patient groups and ethnic groups, as well as their underlying mechanisms, require further investigation.

## Figures and Tables

**Table 1:** Baseline characteristics of study participants.

Characteristic	Total *n* = 4841	Incident Diabetes		*p*-Value
		No *n* = 4004	Yes *n* = 837	
Age (years) *	61.9 (6.6)	62.0 (6.7)	61.4 (6.4)	0.033
Male	2057 (42.5)	1676 (41.9)	381 (45.5)	0.056
Education (years)				0.020
<9	3496 (73.9)	2858 (73.1)	638 (77.8)	
9–11	688 (14.5)	585 (15.0)	103 (12.6)	
≥12	546 (11.5)	467 (11.9)	79 (9.6)	
Smoking status				0.002
Never	2434 (51.9)	2062 (53.0)	372 (46.3)	
Former	1497 (31.9)	1214 (31.2)	283 (35.2)	
Current	760 (16.2)	611 (15.7)	149 (18.5)	
Alcohol consumption				0.001
Abstainer	1333 (30.4)	1067 (29.3)	266 (35.9)	
Moderate	2730 (62.3)	2296 (63.1)	434 (58.6)	
High	317 (7.2)	277 (7.6)	40 (5.4)	
Moderate or high physical activity	1618 (33.5)	1333 (33.4)	285 (34.3)	0.644
Daily vegetable consumption	1707 (36.1)	1420 (36.3)	287 (35.1)	0.537
Daily fruit consumption	2949 (62.8)	2449 (63.0)	500 (61.7)	0.514
Weekly fish consumption	3032 (66.3)	2497 (66.1)	535 (66.8)	0.757
Regular intake of multivitamin supplements	1928 (41.9)	1593 (41.8)	335 (42.0)	0.953
Family history of diabetes	1674 (35.2)	1297 (33.0)	377 (45.5)	<0.001
History of cardiovascular disease	800 (16.5)	627 (15.7)	173 (20.7)	<0.001
History of cancer	359 (7.4)	301 (7.5)	58 (6.9)	0.605
Antihypertensive medication	1937 (40.1)	1516 (37.9)	421 (50.5)	<0.001
Lipid-lowering medication	501 (10.4)	397 (9.9)	104 (12.5)	0.034
Body mass index (kg/m^2^) *	27.4 (4.3)	27.0 (4.1)	29.2 (4.6)	<0.001
HbA_1c_ (mmol/L) *	5.6 (0.4)	5.5 (0.4)	5.8 (0.4)	<0.001
Total cholesterol (mg/dL) *	232.8 (41.9)	232.9 (41.8)	232.6 (42.2)	0.845
HDL cholesterol (mg/dL) *	54.7 (15.1)	55.6 (15.3)	50.1 (13.5)	<0.001
Triglycerides (mg/dL) *	131.9 (77.5)	126.8 (74.0)	156.5 (88.4)	<0.001
C-reactive protein (mg/L) *	4.0 (8.0)	3.9 (8.1)	4.5 (7.1)	0.037
Systolic blood pressure (mmHg) *	138.9 (19.4)	138.5 (19.5)	141.0 (18.9)	0.001
Estimated glomerular filtration rate (mL/min/1.73 m^2^) *	77.4 (18.5)	77.0 (18.5)	79.6 (18.2)	<0.001

* mean ± standard deviation (SD) is reported. *n* (%) refers to the original data without imputation. Abbreviations: HDL: high-density lipoprotein.

**Table 2:** Hazard ratios (95% CI) of developing diabetes by quintiles and per standard-deviation decrease in VDBP, as well as total, “non-bioavailable”, “bioavailable”, and free 25(OH)D concentrations, adjusted for covariates.

	VDBP	Total 25(OH)D	“Non-Bioavailable” 25(OH)D	“Bioavailable” 25(OH)D	Free 25(OH)D
**Median (IQR)**	(µg/mL)	(nmol/L)	(nmol/L)	(ng/mL)	(pg/mL)
Quintile 1	261.7 (23.3)	29.5 (2.9)	25.5 (3.4)	1.2 (0.3)	2.7 (0.8)
Quintile 2	292.3 (12.8)	36.3 (4.2)	31.5 (3.6)	1.7 (0.3)	3.9 (0.5)
Quintile 3	316.4 (12.0)	45.1 (4.4)	39.1 (4.1)	2.2 (0.3)	5.0 (0.6)
Quintile 4	344.0 (16.7)	56.7 (7.8)	49.6 (6.9)	2.9 (0.4)	6.5 (0.9)
Quintile 5	399.4 (52.9)	80.6 (21.4)	69.8 (19.0)	4.4 (1.4)	9.7 (3.3)
**Model 1**					
By quintile					
Quintile 1	1.22 (0.98, 1.53)	1.30 (1.03, 1.65)	1.31 (1.03, 1.65)	1.20 (0.95, 1.51)	1.15 (0.91, 1.44)
Quintile 2	1.10 (0.88, 1.37)	1.20 (0.96, 1.52)	1.26 (1.00, 1.59)	1.12 (0.89, 1.41)	1.14 (0.91, 1.43)
Quintile 3	1.05 (0.84, 1.32)	1.12 (0.89, 1.41)	1.20 (0.95, 1.51)	1.02 (0.81, 1.29)	0.99 (0.79, 1.24)
Quintile 4	1.19 (0.96, 1.49)	1.08 (0.86, 1.36)	1.18 (0.94, 1.48)	1.00 (0.80, 1.25)	0.94 (0.75, 1.18)
Quintile 5	Ref	Ref	Ref	Ref	Ref
Per SD decrease	1.05 (0.97, 1.13)	1.09 (1.01, 1.18)	1.09 (1.01, 1.18)	1.06 (0.98, 1.15)	1.07 (0.98, 1.16)
**Model 2**					
By quintile					
Quintile 1	1.37 (1.09, 1.72)	1.31 (1.03, 1.66)	1.30 (1.02, 1.65)	1.12 (0.89, 1.42)	1.11 (0.88, 1.41)
Quintile 2	1.24 (0.98, 1.55)	1.15 (0.91, 1.45)	1.22 (0.96, 1.54)	1.07 (0.84, 1.34)	1.10 (0.87, 1.38)
Quintile 3	1.13 (0.90, 1.42)	1.08 (0.86, 1.36)	1.16 (0.92, 1.46)	1.01 (0.80, 1.28)	1.00 (0.80, 1.25)
Quintile 4	1.22 (0.98, 1.52)	1.09 (0.87, 1.38)	1.18 (0.94, 1.48)	1.01 (0.81, 1.27)	0.98 (0.78, 1.24)
Quintile 5	Ref	Ref	Ref	Ref	Ref
Per SD decrease	1.09 (1.01, 1.18)	1.09 (1.00, 1.18)	1.09 (1.00, 1.18)	1.05 (0.97, 1.14)	1.05 (0.98, 1.14)

Model 1 adjusted for age; sex; education; smoking and drinking status; vegetable, fruit, and fish consumption; regular intake of multivitamin supplements; body mass index; and season of blood draw. Model 2 additionally adjusted for HbA_1c_, total cholesterol, high-density lipoprotein cholesterol, triglycerides, C-reactive protein, systolic blood pressure, estimated glomerular filtration rate, family history of diabetes, history of cardiovascular disease and cancer, antihypertensive medication, lipid-lowering medication, and season of blood draw. Abbreviations: SD: standard deviation; VDBP: vitamin D-binding protein.

**Table 3:** Adjusted hazard ratios * of developing diabetes by per standard-deviation decrease in total, “non-bioavailable”, “bioavailable”, and free 25(OH)D concentrations by population subgroup.

Subgroup [n_cases_/n_at risk_]	VDBP	Total 25(OH)D	“Non-Bioavailable” 25(OH)D	“Bioavailable” 25(OH)D	Free 25(OH)D
**Total 25(OH)D**					
<50 nmol/L (531/2916)	1.07 (0.97, 1.18)	1.14 (0.87, 1.50)	1.13 (0.87, 1.47)	1.06 (0.88, 1.27)	1.07 (0.89, 1.29)
≥50 nmol/L (306/1925)	1.09 (0.95, 1.26)	1.14 (0.98, 1.31)	1.14 (0.99, 1.32)	1.05 (0.94, 1.17)	1.06 (0.95, 1.18)
p-interaction	0.56	0.78	0.86	0.60	0.54
**Age**					
<65 years (546/3045)	1.04 (0.94, 1.14)	1.09 (0.99, 1.21)	1.09 (0.99, 1.20)	1.08 (0.98, 1.20)	1.09 (0.99, 1.21)
≥65 years (291/1796)	1.19 (1.03, 1.37)	1.09 (0.94, 1.26)	1.10 (0.95, 1.28)	1.01 (0.90, 1.14)	1.02 (0.91, 1.14)
p-interaction	0.16	0.71	0.82	0.38	0.38
**Sex**					
Female (456/2784)	1.08 (0.98, 1.20)	1.20 (1.03, 1.39)	1.20 (1.03, 1.39)	1.10 (0.95, 1.27)	1.11 (0.96, 1.28)
Male (381/2057)	1.10 (0.96, 1.25)	1.06 (0.96, 1.17)	1.06 (0.96, 1.17)	1.04 (0.94, 1.14)	1.04 (0.95, 1.14)
p-interaction	0.34	0.06	0.06	0.20	0.16
**Body mass index**					
<25 kg/m^2^ (139/1448)	0.97 (0.81, 1.16)	1.12 (0.92, 1.36)	1.12 (0.93, 1.35)	1.09 (0.88, 1.34)	1.12 (0.91, 1.38)
≥25 kg/m^2^ (696/3388)	1.13 (1.03, 1.23)	1.11 (1.01, 1.21)	1.11 (1.01, 1.22)	1.07 (0.98, 1.16)	1.07 (0.98, 1.16)
p-interaction	0.31	0.83	0.84	0.76	0.95
Season **					
Winter (544/3130)	1.08 (0.99, 1.19)	1.09 (0.98, 1.22)	1.10 (0.98, 1.23)	1.04 (0.94, 1.15)	1.04 (0.94, 1.15)
Summer (293/1711)	1.09 (0.92, 1.29)	1.08 (0.96, 1.22)	1.08 (0.96, 1.22)	1.08 (0.96, 1.22)	1.09 (0.96, 1.24)
p-interaction	0.96	0.41	0.34	0.96	0.95
**Family history of diabetes**					
No (451/3084)	1.05 (0.95, 1.17)	1.11 (1.00, 1.24)	1.11 (1.00, 1.24)	1.10 (0.98, 1.22)	1.10 (0.99, 1.22)
Yes (377/1674)	1.13 (1.00, 1.28)	1.05 (0.93, 1.19)	1.06 (0.94, 1.20)	1.01 (0.91, 1.12)	1.01 (0.91, 1.12)
p-interaction	0.64	0.57	0.63	0.37	0.41
**Baseline HbA_1c_**					
≤6% (631/4380)	1.07 (0.97, 1.17)	1.15 (1.04, 1.27)	1.15 (1.04, 1.26)	1.13 (1.02, 1.25)	1.13 (1.02, 1.25)
6–6.5% (206/460)	1.09 (0.91, 1.31)	0.97 (0.84, 1.11)	0.97 (0.84, 1.12)	0.96 (0.87, 1.07)	0.98 (0.89, 1.09)
p-interaction	0.73	0.01	0.02	0.01	0.03
**Cardiovascular disease**					
No (664/4041)	1.09 (1.00, 1.19)	1.13 (1.03, 1.24)	1.13 (1.03, 1.24)	1.07 (0.97, 1.16)	1.07 (0.98, 1.17)
Yes (173/800)	1.07 (0.89, 1.29)	0.95 (0.80, 1.14)	0.95 (0.79, 1.13)	0.99 (0.83, 1.19)	1.00 (0.84, 1.19)
p-interaction	0.89	0.43	0.39	0.89	0.88
**Cancer**					
No (779/4482)	1.09 (1.00, 1.18)	1.10 (1.01, 1.19)	1.10 (1.01, 1.19)	1.06 (0.98, 1.15)	1.07 (0.98, 1.16)
Yes (58/359)	1.13 (0.76, 1.69)	1.11 (0.75, 1.64)	1.10 (0.75, 1.63)	1.07 (0.74, 1.53)	1.10 (0.75, 1.62)
p-interaction	0.84	0.60	0.65	0.46	0.48

* The regression models were adjusted for age; sex; education; smoking and drinking status; vegetable, fruit, and fish consumption; regular intake of multivitamin supplements; body mass index; HbA_1c_; total cholesterol; high-density lipoprotein cholesterol; triglycerides; C-reactive protein; systolic blood pressure; estimated glomerular filtration rate; family history of diabetes; history of cardiovascular diseases and cancer; antihypertensive medication; lipid-lowering medication; and season of blood draw. ** winter = November to April; summer = May to October.

## Data Availability

Restrictions due to informed consent apply to the availability of these data.

## Supplementary Materials

The following are available online at https://www.mdpi.com/article/10.3390/nu14163282/s1, Figure S1: Cumulative incidence of type 2 diabetes over the 14-year follow-up according to vitamin D biomarker concentrations, Table S1: Combination of SNP rs7041 and rs4588 for coding VDBP genotype.
