# Supplementary material for: Consistent Inverse Associations of Total, “Bioavailable”, Free, and “Non-Bioavailable” Vitamin D with Incidence of Diabetes among Older Adults with Lower Baseline HbA1c (≤6%) Levels
Source: Nutrients. 2022 Aug 11;14(16):3282. doi: 10.3390/nu14163282 (PMC9413175; doi:10.3390/nu14163282)
Supplement: Supplementary file 1 [file nutrients-14-03282-s001.zip › nutrients-1835594-supplementary.pdf]

Supplementary Materials

Supplementary Methods

Measurement and standardization of total 25(OH)D concentrations

In 2006, we measured serum 25(OH)D concentrations among women by using the automated Diasorin-Liaison analyzer (Diasorin Inc) in the central laboratory of the University Clinic Heidelberg (Heidelberg, Germany). The analyzer has a within-assay coefficient of variation (CV) of 8-21% and a between-assay CV of 8-34%. The lower detection limit was 15 nmol/L. In 2009, we measured serum 25(OH)D concentrations among men by using the automated IDS-iSYS analyzer (Immunodiagnostic Systems GmbH) in the laboratory of the Institute for Experimental Endocrinology, Charité University Medicine (Berlin, Germany). The assay has an intraassay CV <7.3%, and an interassay CV <8.9%. The lower detection limit was 9 nmol/L. We standardized both immunoassays to the gold standard method LC-MS/MS, to reduce potential bias aroused from two different measurement methods. Serum samples of 100 males and females from each assay were randomly extracted and re-measured by using isotope-dilution LC-MS/MS in 2011 in the Department of Clinical Chemistry, Canisius Wilhelma Hospital (Nijmegen, Netherlands). Because NIST SRM standard was not available at that time, the human serum calibrator of Chromsystem, Munich, Germany for LC-MS/MS standardization was used. We excluded three pairs of influential outliers from each assay because they were >2 standard deviations of the mean assay difference. Therefore, 97 pairs of samples were included in the standardization analysis. There were high correlations between measurements with the Diasorin-Liaison analyzer and LC-MS/MS, and between the IDS-iSYS analyzer and LC-MS/MS. The Spearman's rank correlation coefficients were 0.83 and 0.86, respectively. We observed that values measured by measured with LC-MS/MS were higher than those of the Diasorin-Liaison analyzer. Ordinary least-squares linear regression models were conducted and used to fit results for 25(OH)D concentration standardization. We used the following equations for serum 25(OH)D measured with the Diasorin-Liaison analyzer:

25(OH)D (LC-MS/MS) [nmol/L]=0.7989 * 25(OH)D (Diasorin-Liaison analyzer) [nmol/L] + 17.58 nmol/L

or with the IDS-iSYS analyzer:

25(OH)D (LC-MS/MS) [nmol/L]=0.9526 * 25(OH)D (IDS-iSYS analyzer) [nmol/L] – 0.3222 nmol/L

Genotyping and imputation of single nucleotide polymorphisms (SNPs) rs7041 and rs4588

During the health check-up, blood samples were collected by general practitioners and then sent to the central laboratory for storage at −80 °C until analysis. Based on whole blood samples, we extracted deoxyribonucleic acid (DNA) by using a salting out procedure. We performed genotyping by using the Illumina Infinium OncoArray and Global Screening Array BeadChips (Illumina, San Diego, CA, USA). We pre-phased with SHAPEIT software v2.12, and used IMPUTE2 v2.3.2 to impute genotypes for common variants across the genome by using data from 1000 Genomes Project (phase 3, Oct. 2014). All genomic locations are given in NCBI Build 37/UCSC hg19 coordinates. We excluded SNPs with an information metric I < 0.70, and those with a minor allele frequency (MAF) < 1%. We extracted SNPs of rs7041 and rs4588 by using PLINK v1.90, and coded VDBP genotypes (**Table S1**).

Table S1. Combination of SNP rs7041 and rs4588 for coding VDBP genotype

rs7041 genotype	rs4588 genotype	VDBP genotype
TT	CC	GC1f-1f
TG	CC	GC1f-1s
TT	CA	GC1f-2
GG	CC	GC1s-1s
TG	CA	GC1s-2
TT	AA	GC2-2

Abbreviations: VDBP: vitamin D-binding protein.

Figure S1. Cumulative incidence of type 2 diabetes over the 14-year follow-up according to vitamin D biomarker concentrations

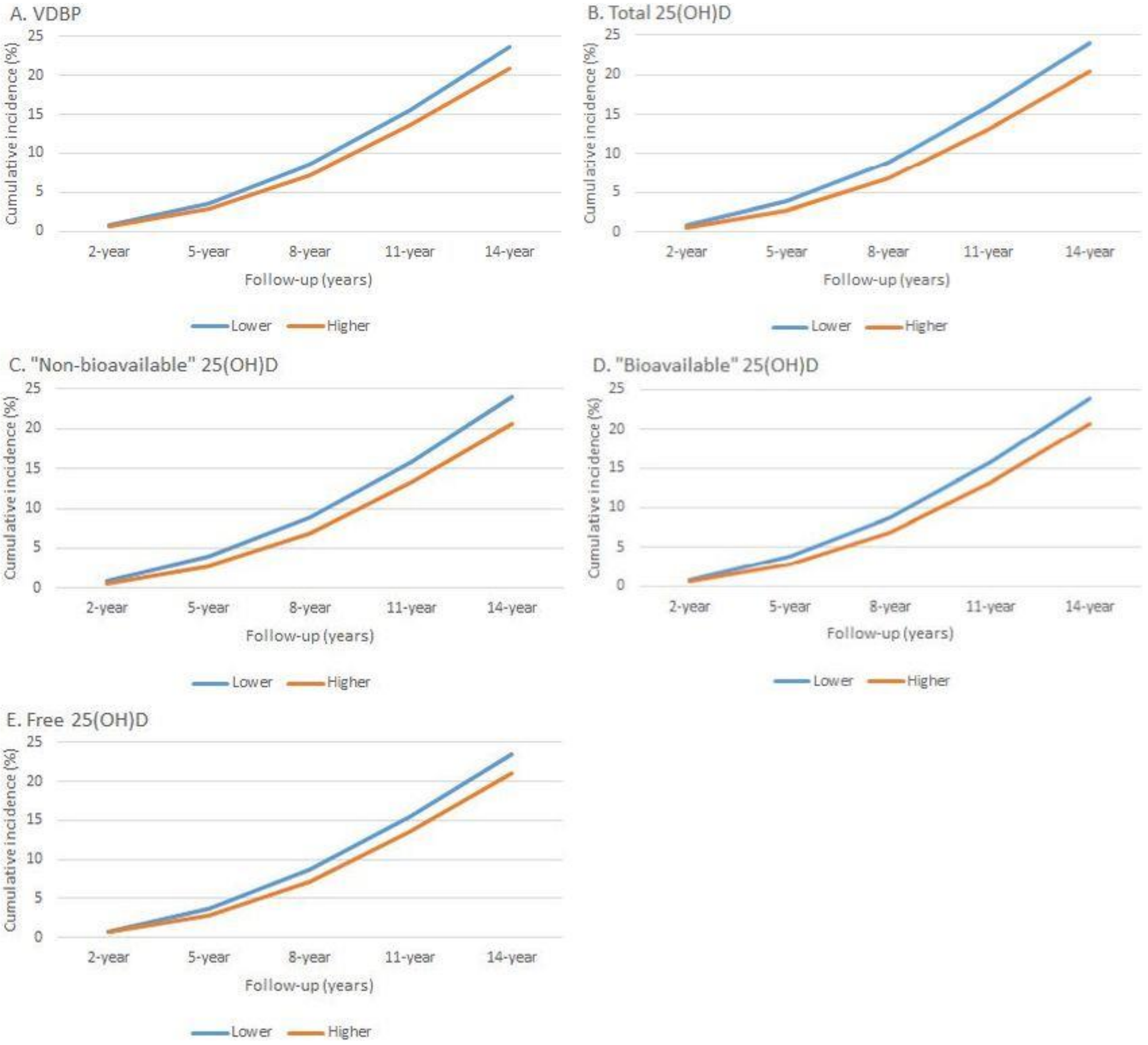

The cut-off points between lower and higher levels were the median values of VDBP (316.3 µg/mL), total (45.1 nmol/L), “non-bioavailable” (39.1 nmol/L), “bioavailable” (2.2 ng/mL), and free 25(OH)D (5.0 pg/mL) concentrations. Abbreviations: VDBP: vitamin D-binding protein.
